# Delayed Postanoxic Encephalopathy Following Acute Carbon Monoxide Poisoning: A Case Report and Literature Review

**DOI:** 10.7759/cureus.84024

**Published:** 2025-05-13

**Authors:** Mina Aallam, Nada Nfaoui, Yasmina Zakaria, Mohamed Chraa, Nissrine Louhab

**Affiliations:** 1 Neurology, Mohammed VI University Hospital Center, Marrakesh, MAR; 2 Neurology, Mohammed VI University Hospital Center, Cadi Ayyad University, Marrakesh, MAR

**Keywords:** brain mri, carbone monoxide poisoning, dns, hbot (hyperbaric oxygen therapy), neurocognitive

## Abstract

Carbon monoxide (CO) poisoning is the leading cause of death from accidental poisoning worldwide. It is associated with significant mortality and morbidity. The mere notion of exposure to risk should lead to the diagnosis of CO poisoning in an emergency situation. Neurological (coma and delayed neuropsychological sequelae) and cardiac (ischemic changes with normal coronary arteries) clinical scenarios are explained by complex mechanisms: carboxyhemoglobin formation, cytochrome oxidase inhibition, oxidative stress, as well as ischemia-reperfusion phenomenon. Delayed neurological sequelae (DNS) or delayed encephalopathy is characterized by a neurological clinical picture that arises after acute CO intoxication and after a period of apparent recovery. The onset of DNS is unpredictable. The true prevalence of DNS is difficult to determine; the estimates range from 1% to 47% of patients after CO poisoning. The exact incidence rate is also unclear. Studies using rigorous methodologies, including neuropsychological testing, report the frequency to be as high as 67%. CO poisoning causes severe neurocognitive after-effects that are rarely studied in the literature.

Here, we describe the case of a patient aged 18 years old with no known comorbidities, a victim of accidental CO poisoning, who initially presented with behavioral disorders. The evolution was marked by the occurrence of memory and attention disorders a few weeks later. Neuropsychological assessment revealed a dysfunction of several processes (executive, attentional, and memory). Brain MRI revealed restrictive bilateral occipital, frontal, and temporal lesions of hypoxic origin.

The patient underwent three sessions of hyperbaric oxygen therapy and received symptomatic drug treatment and neurocognitive rehabilitation, with a more or less favorable evolution of her disorders.

## Introduction

Carbon monoxide (CO) is an odorless, colorless, and tasteless gas, rendering its detection difficult. At concentrations higher than 35 parts per million (ppm), it is toxic to humans. CO poisoning is a leading cause of mortality due to poisoning in many countries, with data suggesting this may account for over half of all fatal poisonings in the world [1]. CO is the product of the incomplete combustion of hydrocarbon fuels and can be produced in fires, stoves, and internal combustion engines, among others. Typically, patients with CO poisoning present to the emergency department with acute nonspecific symptoms that completely revert after oxygen therapy [2]. However, some of these patients develop delayed neurological sequelae (DNS). The latter amend slowly and are not a direct cause of death, though they may cause significant morbidity and healthcare spending. The spectrum of DNS includes motor impairment with loss of autonomy in performing instrumental tasks of daily living, as well as significant cognitive, psychological, and mental health challenges. There are also economic impacts on patients, families, and society at large. Currently, the role of hyperbaric oxygen therapy (HBO) for the treatment of CO poisoning and the prevention of DNS remains controversial [3]. Our case illustrates the neurocognitive sequelae following CO poisoning and their management in light of the literature.

## Case presentation

A previously healthy 18-year-old female North African presented to the emergency department after four hours of CO exposure due to a malfunctioning water heater. On admission, she was unconscious, with a Glasgow Coma Score of 7/15 (eye opening response: 2, verbal response: 1, motor response: 4), necessitating intubation and mechanical ventilation. Laboratory investigations showed a carboxyhemoglobin level of 18% (normal ≤ 3%), elevated serum lactate at 6.8 mmol/L (normal range: 0.5-1.5 mmol/L), and elevated troponin T level at 265 ng/L (normal ≤ 16ng/L) (Table 1). The rest of the biological assessment was normal (blood count, renal and hepatic assessment), and the electrocardiogram results were within normal limits.

**Table 1:** Laboratory findings on carboxyhemoglobin (COHb), serum lactate, and troponin T

Lab	Result	Reference range
Carboxyhemoglobin	18%	<3%
Serum lactate	6.8 mmol/L	0.5-1.5
Troponin T	265 ng/L	<16

Initial management included oxygenation via mechanical ventilator, sedation, fluid therapy, and correction of electrolyte imbalances (correction of lactic acidosis and normalization of carboxyhemoglobin). The patient underwent three HBO sessions at two atmospheres in a specialized center, resulting in partial neurological improvement (the patient improved her Glasgow score to 13/15, subsequently extubated). The patient was subsequently transferred to the neurology department for further management. Upon examination, she exhibited behavioral agitation, shouting, memory disturbances, and executive dysfunction. Neurological assessment revealed impaired higher cognitive functions with Mini-Mental State Examination (orientation: 5; attention and calculation: 0; recall: 0; praxis 0; language: 7; registration: 3) and Montreal Cognitive Assessment (orientation: 5; attention and calculation: 0; delayed recall: 0; visuospatial/executive: 1; abstraction: 1; naming: 3; attention: 3; language: 2) scores of 15/30 each.

**Figure 1:**
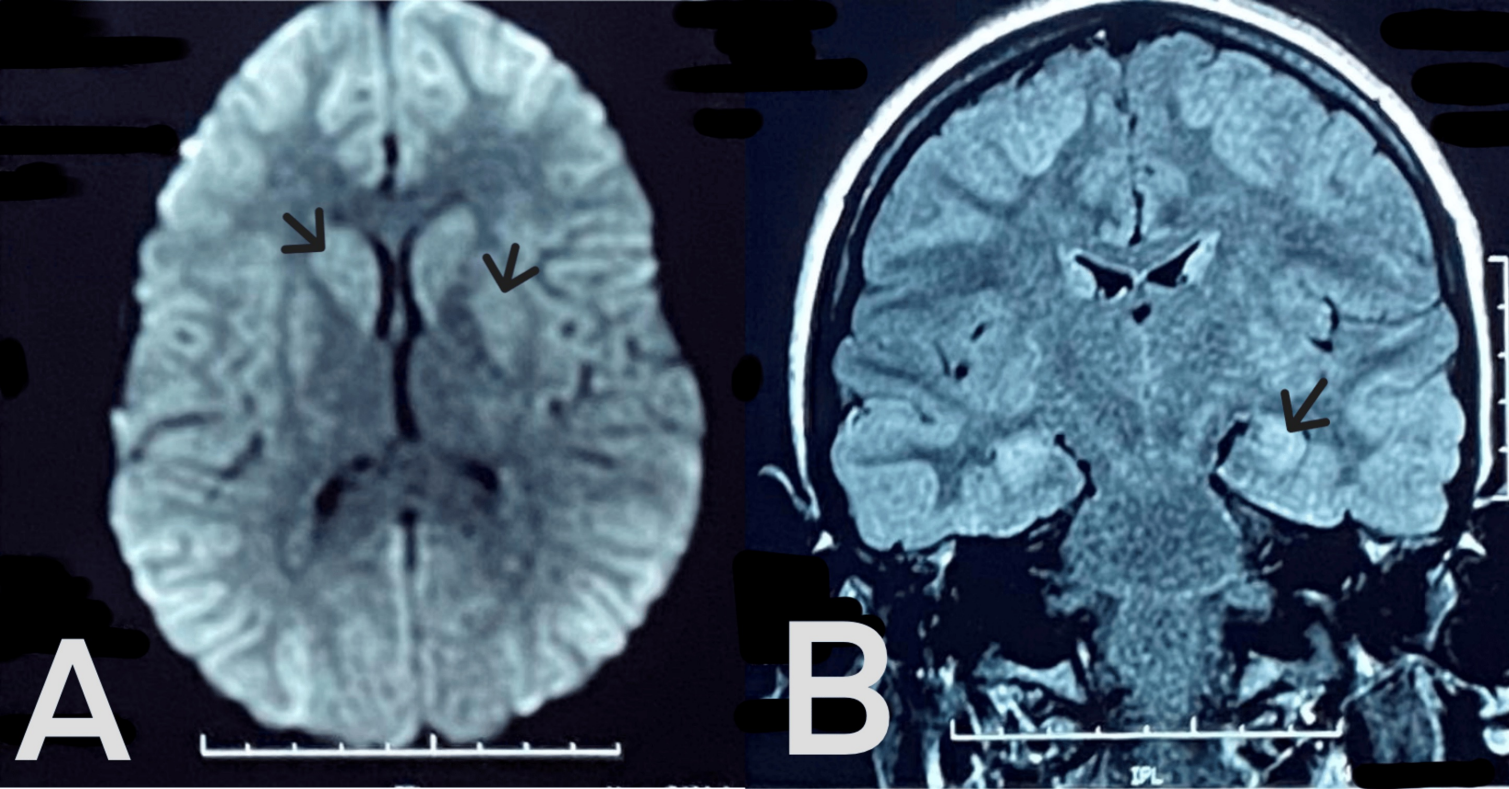
Admission brain magnetic resonance imaging (MRI) (axial and coronal view). (A) Restriction in diffusion sequence on bilateral temporal, occipital, frontal, putamen, and caudate nucleus. (B) Bilateral symmetrical abnormal signal intensity involving the mesial temporal lobe, occipital, and frontal lobes, showing high signal intensity (red arrow) on T2 Fluid-Attenuated Inversion Recovery (FLAIR).

Brain magnetic resonance imaging (MRI) demonstrated bilateral occipital, frontal, and temporal hypoxic lesions, including bilateral caudate nucleus and putamen involvement (Figure 1). She was started on escitalopram and low-dose anxiolytics, which led to partial behavioral improvement over an eight-week period. Persistent cognitive deficits prompted a detailed neuropsychological evaluation 10 months after exposure. The evaluation revealed significant impairments in executive function (inhibition, multitasking, flexibility), attentional control, and hippocampal memory (including working and episodic memory). The patient also exhibited impulsiveness associated with disinhibition. The patient discontinued her academic activities and began a structured neurocognitive rehabilitation program with a relative improvement in calculation abilities and practical functions, but still suffers from memory disorders, impulsiveness, and attention disorders.

## Discussion

CO poisoning is prevalent in Morocco [4]. With an incidence of 137 cases per million and a mortality of 4.6 deaths per million, CO poisoning is an important cause of intoxication worldwide. The incidence of CO poisoning has remained unchanged over the past 25 years; however, the mortality rate has decreased by 36% [5].

CO poisoning is diagnosed on clinical grounds in a person with proven or probable exposure to CO. Although carboxyhemoglobin levels in the blood may be increased, normal levels do not rule out the diagnosis in the context of a consistent history and symptoms.

CO poisoning results in altered oxygen-carrying capacity of red blood cells as well as impairment of the cellular respiratory chain and immunomodulatory processes. These may result in cellular dysfunction and cell death in brain and myocardial cells. Even when carboxyhemoglobin levels have reduced, cells remain at risk of dysfunction. Thus, clinical symptoms do not necessarily correlate with the level of carboxyhemoglobin or its elimination [6]. The spectrum of clinical symptoms of acute CO poisoning includes headache, dizziness, altered consciousness, and angina. Acute poisoning may lead to death. The clinical manifestation depends on the concentration of the gas and duration of exposure [7]. At present, no consensus exists as to diagnostic criteria or treatment [8]. Neurological manifestations, which occur mainly when carboxyhemoglobin levels exceed 25%, may either have an immediate or delayed onset. When symptoms occur immediately, patients may recover progressively over months. At times, patients retain some neuropsychiatric sequelae, termed persistent neurological sequelae (PNS). When symptoms are delayed in onset, they occur within six weeks after initial full recovery. These delayed neuropsychiatric sequelae (DNS) may be encountered up to six years after CO poisoning. PNS and DNS are due to hypoxic brain injury, inflammation, free radical generation, and apoptosis. These sequelae include a range of cognitive impairment, depression, anxiety, vestibular and motor deficits [1], urinary and fecal incontinence, gait disturbance, Parkinson-like syndromes, and mutism [9].

According to Mimura et al., cognitive impairment was found in 68.6% of patients, while neurological sequelae were present in 48.7% of patients 33 years after CO poisoning [1].

Management decisions regarding CO poisoning should be individualized, as guidelines for the treatment are still under development [10]. Extraction of patients from the offending source is the initial step in limiting damages. Therapy aimed at reversing tissue hypoxia and removing CO from the body should be initiated promptly [11]. HBO is the main approach to achieving the latter objectives. This therapy entails administering 100% oxygen to the patient in a pressurized chamber in order to increase circulating oxygen and tissue oxygenation. This approach removes CO from hemoglobin, restores oxygen supply at the cellular level, and reduces the damage caused by hypoxia. HBO has been shown to be effective in preventing DNS [5].

In Morocco, a study of 309 patients with acute CO poisoning who underwent HBO revealed the overall positive outcomes for patients. However, in 23% of cases, minor or major sequelae persisted. This study points to the importance of a timely administration of HBO [12]. However, in a systematic review of the Cochrane database by Smollin and Olson, HBO was not found to reduce the incidence of neurological symptoms in patients with CO poisoning [13]. Evidence-based recommendations regarding the exact parameters of HBO, including the optimal atmospheric pressure as well as the duration and number of sessions, are lacking. Furthermore, other approaches, such as hypothermia and sympatholytics, may be promising therapies in CO poisoning, as tissue hypoxia and catecholamine crisis are the underlying mechanisms of DNS. The administration of hypothermia with sedation may help prevent DNS in patients with CO poisoning [11].

Antioxidants, such as N-acetyl cysteine, pulse steroids, and erythropoietin, have uncertain benefits. The prognosis is enhanced if prompt HBO is not used as a stand-alone therapy [3]. Supportive therapy, including physical rehabilitation and cognitive therapy as well as drug treatment, is crucial [5].

Data on the prognosis of CO poisoning are inconclusive. However, it is agreed that outcomes are predicated on factors linked to CO exposure and to individual risk factors. The quantity and duration of CO exposure, as well as the age of the patient, the presence of comorbid conditions, and pregnancy are important determinants of prognosis [13].

## Conclusions

CO poisoning remains a major public health problem in Morocco and may be encountered both in household and professional environments. The presence of significant neuropsychiatric sequelae as well as high mortality underscores the need for prompt diagnosis and appropriate management. Outcomes are variable and conditioned by factors associated with exposure as well as factors intrinsic to patients and the administration of early HBO. This case reiterates the need for early recognition of CO poisoning. Further research is needed to better understand the mechanisms underlying neurocognitive deficits, as well as to establish consensus guidelines on diagnosis and treatment. Effective rehabilitation strategies are also needed. Importantly, awareness should be raised on the risks of CO poisoning so as to ensure appropriate preventive measures are put in place.
